# Development of CE-C^4^D Method for Determination Tropane Alkaloids

**DOI:** 10.3390/molecules26195749

**Published:** 2021-09-22

**Authors:** Małgorzata Gołąb, Martyna Przybyłowska, Petr Kubáň, Petra Itterheimová, Michał Woźniakiewicz

**Affiliations:** 1 Laboratory for Forensic Chemistry, Department of Analytical Chemistry, Faculty of Chemistry, Jagiellonian University, 30-387 Krakow, Poland; mgolab@doctoral.uj.edu.pl (M.G.); martyna.przybylowska@student.uj.edu.pl (M.P.); 2 Department of Bioanalytical Instrumentation, Institute of Analytical Chemistry, Czech Academy of Sciences, 60200 Brno, Czech Republic; petr.kuban@gmail.com (P.K.); p.itterheimova@gmail.com (P.I.); 3 Central European Institute of Technology and Faculty of Science, Masaryk University, 62500 Brno, Czech Republic

**Keywords:** capillary electrophoresis, tropane alkaloids, atropine, scopolamine, CE-C^4^D, conductivity detection

## Abstract

A fast method for the determination of tropane alkaloids, using a portable CE instrument with a capacitively coupled contactless conductivity detector (CE-C^4^D) was developed and validated for determination of atropine and scopolamine in seeds from *Solanaceae* family plants. Separation was obtained within 5 min, using an optimized background electrolyte consisting of 0.5 M acetic acid with 0.25% (*w/v*) β-CD. The limit of detection and quantification was 0.5 µg/mL and 1.5 µg/mL, respectively, for both atropine and scopolamine. The developed method was validated with the following parameters—precision (CV): 1.07–2.08%, accuracy of the assay (recovery, RE): 101.0–102.7% and matrix effect (ME): 92.99–94.23%. Moreover, the optimized CE-C^4^D method was applied to the analysis of plant extracts and pharmaceuticals, proving its applicability and accuracy.

## 1. Introduction

Atropine (Atr) and scopolamine (Sco) are tropane alkaloids found in high concentrations in *Solanaceae* (nightshades) plants, such as *Datura* L., *Brugmansia* Pers., *Atropa* L., *Scopolia* Jacq. [1]. Atropine is used for medicinal purposes mainly in case of arrythmias, organophosphate poisonings or prior surgical procedures providing sedative effects. Scopolamine shows also memory impairment effects and in case of abdominal cavity or bladder pain as well as in a motion sickness is used in the form of scopolamine-*N*-butyl bromide [2]. Due to the effects of atropine and scopolamine on the central nervous system, the above-mentioned plants are also sometimes used for recreational purposes leading to hallucinations and agitation, which are often associated with their overdose. The characteristic symptoms of overdose are dry mouth with burning sensation, difficulty swallowing, photophobia, redness and dry skin, increased body temperature, rash, nausea and vomiting, tachycardia and increased blood pressure, somnolence and, finally, respiratory and circulatory failure, which may be fatal [3,4,5]. Compared to atropine, scopolamine has a much more pronounced effect on the central nervous system, causing drowsiness and dementia even in small doses [1]. The development of methods for determination of atropine and scopolamine in plant material, pharmaceuticals or biological samples is, thus, of high importance.

Over the past few years, a variety of analytical methods were developed to determine tropane alkaloids in plant material or biological samples, mostly high-performance liquid chromatography [3,4,5,6,7,8,9] and gas chromatography [10,11,12]. However, these techniques are quite expensive in terms of equipment and required chemicals. Capillary electrophoresis (CE) was also used for analysis of tropane alkaloids with electrochemical or spectrophotometric [13,14,15,16,17] detection, including micellar electrokinetic capillary chromatography (MEKC) [18,19]. Only a few publications utilized capacitively coupled contactless conductometric detector (C^4^D) [20,21].

Within a framework of this research, ultrasound-assisted extraction (UAE) was used for sample preparation, because it significantly speeds up the extraction process. UAE also allows using less organic solvent compared to other extraction techniques, making this approach fit well within the principles of “green analytical chemistry”. Atropine, scopolamine and other matrix components were detected using CE-C^4^D. C^4^D is widely used in portable systems because it can be miniaturized still providing better sensitivity than the UV-Vis detector. The operation of the C^4^D is based on measuring the conductivity differences between background electrolyte (BGE) and the migrating zones of separated analytes [22,23]. This might be found as an advantage in comparison with commonly used spectrophotometric detection as it enables to detect all ionized species in the sample, such as inorganic anions, metal ions or non-aromatic organic compounds at the same time with aromatic organic compounds (e.g., amino acids), which cannot be achieved by a UV-Vis detector due to the low absorbance of these species. However, the use of C^4^D could be challenging in analysis of compounds with similar structure and physiochemical properties due to very close mass to charge ratio and requirement of low conductivity BGE.

The aim of this research was to develop a simple, cost-effective and fast method for determination of atropine and scopolamine by the CE-C^4^D system. The developed method should provide sensitive and selective analytical results, derived from excellent analytical performance of the C^4^D detection system comparing to UV-Vis detection and high separation power offered by capillary electrophoresis, respectively. Additionally, the effectiveness of the method was evaluated using the RGB method algorithm, which provides a concept for ranking and comparing different analytical methods [24,25].

## 2. Results and Discussion

### 2.1. Optimization of Separation Conditions

#### 2.1.1. Selection of the Background Electrolyte

Atropine and scopolamine are tropane alkaloids with very similar structure. However, they differ in *pK_a_*/logP (partition coefficient) values, which are 9.43/1.83 and 7.75/0.98 for atropine and scopolamine, respectively. One possibility to separate them by the capillary zone electrophoresis is to use BGE with pH close to *pK_a_* value of scopolamine, where the analytes will have different net charge and the mass to charge ratio will be different enough to separate them. Such an approach is, however, not robust, as it has been shown by Saiz et al. [20], because it is very pH dependent and a tiny change in pH results in an incomplete separation. To achieve a fast analysis, stable baseline and good intensity of the analytical signal, we started with optimization of the BGE composition and pH. Four BGEs at different pH values were tested: (A-2.5) 0.5 M acetic acid (pH = 2.5); (A-3.1) 0.025 M acetic acid (pH = 3.1); (HA-4.1) 0.005 M L-histidine (HIS), 0.025 M acetic acid (pH = 4.1) and (HM-6.1) 0.02 M HIS, 0.02 M 2-(*N*-morpholino)ethanesulfonic acid (MES) (pH = 6.1).

From the four candidate BGEs, the best results were obtained for the A-2.5 and A-3.1; however, for BGE A-2.5, the analysis time was 1 minute shorter and its buffering capacity was significantly higher than that of A-3.1 (although for all BGEs they were relatively low: see Table S1 in Supplementary Materials). The HM-6.1 BGE had the highest buffering capacity and ionic strength. The separation in this BGE was very fast compared to A-2.5 (1.8 min vs. 4.3 min) but, simultaneously, the detection sensitivity was considerably lower (95% lower than in A-2.5). In the case of HA-4.1 BGE, a stable baseline was not obtained and the peak shapes indicated that this BGE is not suitable. Although none of the above listed buffers enabled complete separation of atropine and scopolamine, A-2.5 BGE was chosen for further investigations, as it provided stable baseline, good sensitivity and short analysis time.

The improvement of electrophoretic separation may be achieved by addition of cyclodextrins (CD). Asztemborska et al. [26] used native cyclodextrins (α, β and γ) and their non-ionic derivatives to investigate complex formation with Atr and Sco. The results indicate that the separation of particular enantiomers of Atr and Sco is challenging, but CDs may be used to increase the separation ability of the BGEs. In the cited work, the authors did not perform the actual separation of these tropane alkaloids, but they determined the effective mobilities in electrolytes containing various CDs. Thus, in the following step the addition of five different cyclodextrins: β-CD, 2-hydroxypropyl-β-CD (HP-β-CD), 2-hydroxypropyl-γ-CD (HP-γ-CD), 2-hydroxyethyl-β-CD (HE-β-CD), heptakis(2,3,6-tri-O-methyl)-β-CD (TM-β-CD), in a concentration 1% (*w/v*) was taken into consideration.

The use of four out of the five tested nonionic CDs resulted in satisfactory separation of target compounds; only for TM-β-CD, an incomplete separation of Atr and Sco was obtained. In comparison to the BGE containing HP-β-CD, the BGEs containing β-CD and HE-β-CD enabled separation with good resolution, but in a shorter time (9 min and R ~ 5.4 vs. 6.5 min and R ~ 3.8 vs. 5.5 min and R ~ 2.5, respectively). The separation of the Atr and Sco with HP-γ-CD was also possible, but the resolution was much lower (R ~ 1.5 within 4.5 min). To minimalize the possibility of Atr and Sco co-migration, while ensuring short analysis time, β-CD and HE-β-CD were selected for further testing.

Because the cyclodextrins are generally quite expensive (i.e., HE-β-CD), further experiments were aimed at decreasing the CD concentration to a minimum, while still maintaining an adequate peak resolution.

**Figure 1:**
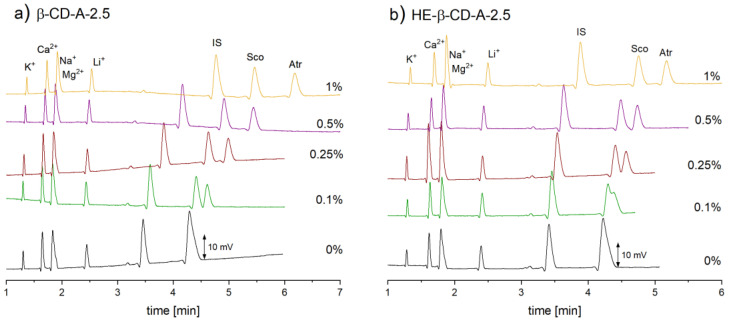
Effect of cyclodextrin concentration (*w/v*) on the separation. BGE: 0.5 M acetic acid with (**a**) β-CD or (**b**) HE-β-CD. Electropherograms for the analysis of standard solution of 25 μg/mL Atr and Sco with 20 μg/mL procaine (IS) and ~2 μg/mL inorganic cations (K^+^, Na^+^, Ca^2+^, Mg^2+^, Li^+^). CE conditions: capillary length 29.9 cm (18.4 cm to the detector); applied voltage: +11 kV; hydrodynamic injection (siphoning) 7 cm height for 30 s.

It was found that for β-CD, it is sufficient to use 0.25% (*w/v*) while for HE-β-CD 1% (*w/v*) concentration was needed. Therefore, 0.5 M acetic acid with 0.25% (*w/v*) β-CD was selected as the optimal BGE. Moreover, the reduction of β-CD concentration in the separation buffer resulted in the reduction of the analysis time by more than 1 min with good resolution (R ~ 2.5, see Figure 1).

**Figure 2:**
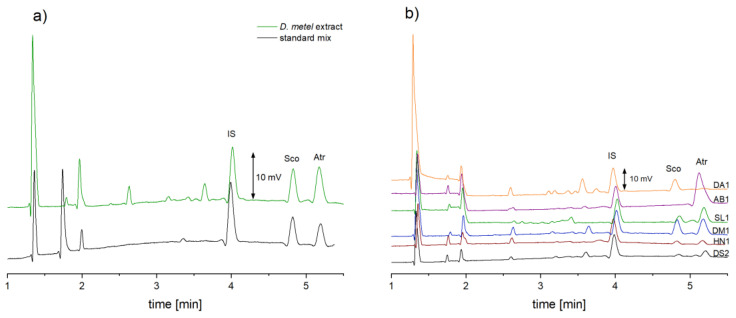
Electropherograms for the analysis of (**a**) mix of standards and *D. metel* seeds extract, (**b**) extracts of seeds (see Table 1): *D. stramonium* (DS2), *H. niger* (HN1), *D. metel* (DM1), *S. lurida* (SL1), *A. belladonna* (AB1), *D. arborea* (DA1). CE conditions: capillary length 29.9 cm (18.4 cm to the detector); applied voltage: + 11 kV; hydrodynamic injection (siphoning) 7 cm height for 30 s; BGE: 0.5 M acetic acid + 0.25% (*w/v*) β-CD.

Since the developed method was intended for analysis of the plant extracts, it was necessary to evaluate whether the methanol concentration and matrix components (e.g., amino acids) influence the migration times of the analytes. The experiments showed that the methanol content in the sample (it was a component of the extraction solvent) had a greater impact on the shifts of migration times than the matrix components present in the extracts. Therefore, for the preparation of standard solutions, the same methanol concentration was used as was for the analyzed extract samples, i.e., 35% (*v/v*). As it can be seen in Figure 2a, adding 35% (*v/v*) of methanol resulted in very good compliance of migration times for the analyzed standard solution and plant material extract.

#### 2.1.2. Instrumental Separation Conditions

In the subsequent part of the optimization of the experimental conditions, we aimed to decrease the analysis time as much as possible. This would allow the performance of more measurements per day and reduce the costs of a single analysis.

**Figure 3:**
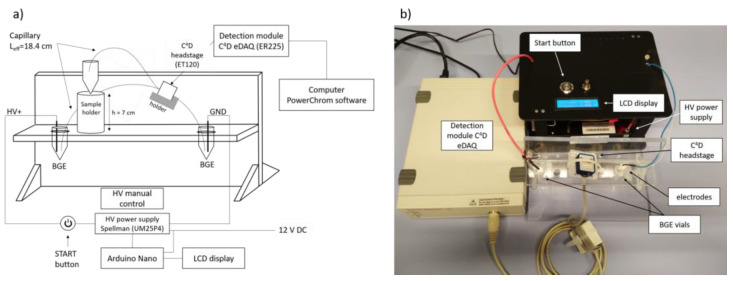
Scheme **(a)** and a photograph **(b)** of the CE-C^4^D in-house built instrument.

The electrophoretic migration velocity depends on the electric field and can be increased by using higher separation voltage or shorter separation capillary. In the constant electric field, the migration time can be manipulated by changing the distance from the inlet to the detector (effective length). The effect of these instrumental parameters on the separation and analysis time was investigated. Shortening the effective length from 18.4 to 9.5 cm (total capillary length was not reduced and it was 29.9 cm) resulted in halving the analysis time while maintaining good resolution (see Figure S1 in the Supplementary Materials). However, due to the design of the CE system (see Figure 3b) the effective length was kept at 18.4 cm, because with short effective capillary length, repeatable injection sequence was not possible.

Another parameter that could be easily modified was the applied high voltage (in the range 11–15 kV), but due to the absence of capillary thermostating, a voltage of +11 kV was selected as the best compromise to keep the Joule heat generation low.

### 2.2. Method Validation

The developed method was validated using the BGE consisting of 0.5 M acetic acid and 0.25% (*w/v*) β-CD, the separation capillary (i.d., 50 μm, o.d. 365 μm) of 29.9 cm total length and 18.4 cm effective length, the voltage of +11 kV, normal polarity and manual sample injection by elevating the injection capillary end to the height of 7 cm for 30 s.

The developed method is characterized by a linear dependence of the analytical signal (peak area for analyte divided by peak area for internal standard (IS)—procaine) on the concentration in the range of 5–50 μg/mL. The R-squared for both atropine and scopolamine was ≥0.996.

The limit of detection (LOD) and limit of quantification (LOQ) were determined by analyzing standard solutions with decreasing concentration of the analytes (see Figure S2 in Supplementary Materials). LOD and LOQ for both, atropine and scopolamine, were 0.5 and 1.5 μg/mL, respectively. All determined validation parameters are listed in Table 2.

Precision was expressed as a coefficient of variation of the calculated concentration of atropine and scopolamine. Intermediate precision was obtained within 2 days (*n* = 5, each day). As it is shown in Table 2, precision was found to be better than 2.1% and intermediate precision was better than 3% at all concentration levels.

The recovery and the matrix effect were determined at two concentration levels and due to the lack of an available matrix without analytes, *D. metel* seed extracts were prepared accordingly. In the case of recovery, the Atr and Sco aliquots were added to the sample before the extraction process, while for the matrix effect evaluation they were added to the extracts prior to the analysis using the CE-C^4^D method. The obtained recovery values were in the range of 101.0 to 102.7%, while the matrix effect was in the range of 92.99–94.23%. These values comply with the recommended level of acceptance, which is 95.0–105.0% and 85–115% for recovery and matrix effect study, correspondingly [27,28].

### 2.3. Analysis of Solanaceae Plant Samples

The developed method was employed for analysis of seeds from *Solanaceae* plants. The obtained results show that there are differences in the content of Atr and Sco in various plants and that there are unidentified matrix components in the studied plants of the genus *Solanaceae*. Further experiments are needed to investigate, whether this method might be used also for the discriminant analysis. As it can be seen in Figure 2b, the analyte peaks are well separated from the matrix components. In addition, all analyzed samples were subjected to quantitative analysis, the concentrations of Atr and Sco were calculated from the calibration curve. Only in two plant extracts (*D. stramonium* and *A. belladonna*), scopolamine was not determined (Table 1).

### 2.4. Analysis of Pharmaceuticals

In order to additionally demonstrate the applicability of the developed method for the determination of atropine, the CE-C^4^D analysis was performed in two pharmaceutical samples. These two samples differed both in the concentration of atropine and in the presence of excipients. Two preparations were selected for the analysis: an eye drop solution (10 mg/mL Atr) and an injection solution (1 mg/mL Atr). Both solutions were diluted with ultrapure water (spiked with IS), so that the final concentration of atropine was 10 µg/mL and IS was 20 µg/mL. For the analysis, three separate samples of each solution were prepared and each one was measured in triplicate.

For the eye drop solution the calculated concentration was 9.76 mg/mL (SD = 0.10, *n* = 9) and for the injection solution was 0.96 mg/mL (SD = 0.01, *n* = 9). The obtained results show good compliance with the declared values. For all the samples relative error did not exceed 5%.

### 2.5. Assesment of the Optimized Method with RGB Model

The developed method was assessed with the RGB model. This model is a new concept for ranking and comparing analytical methods inspired by a common model of colors. In this model, the primary colors describe the main attributes of a method: analytical performance (Red), ecofriendliness and safety for users (Green) as well as productivity and practical aspects (Blue). Moreover, it includes one more quantitative parameter-method whiteness, which is a result of user-defined significance of the primary colors [24,25]. The detailed results are included in Supplementary materials—excel spreadsheet.

Five different methods: CE-C^4^D [20] and this work, GC-MS [10], HPLC-DAD [12] and CE-DAD [15] were evaluated. The analytical performance was found excellent for GC-MS (above 102 points); however, our developed method was very close to this value (97.5). The CE-C^4^D method described previously [20] was assessed with lower marks due to the lack of information about accuracy and recovery, while the HPLC-DAD method was assessed somehow lower (62.5) due to the relatively high detection limit.

Capillary electrophoresis is one of the greenest separation techniques. It requires only limited amount of solvents and chemicals, as in the case of the developed system 2 mL of BGE enables running four analyses (additional 0.5 mL of BGE for capillary rinsing was used for a whole working day). Short analysis time and low energy consumption (ca. 0.008 kWh per analysis) designate the developed system as very effective. It was also found that only 1 mL of extraction solvent can provide efficient isolation of target analytes which also supports “green analytical chemistry” principles by reducing reagent consumption and simultaneously—disposals generation. In this comparison, both CE-C^4^D methods were assessed as representing ecofriendly approaches, with a green value of ≥85; however, our developed instrument was assessed as being riskier due to the missing shield additionally protecting a user from the exposure to high voltage. Surprisingly, the GC-MS method resulted in lower mark (ca. 67), mainly because of significantly higher number of pictograms referring to hazardous materials, which made this method comparable to HPLC-DAD (also ca. 67).

The practical side of the methods (blue value) is affected by several factors (e.g., cost and time efficiency, sample consumption, skills required, miniaturization, integration and automation and portability). In the case of CE-C^4^D methods, these factors balance nicely, resulting in more than 80 points, with the superiority of the method developed by Saiz et al. [20], mainly due to the advanced portability of their instrument. A relatively long time of analysis and the minimum chance for portability in the case of HPLC-DAD put this method at the low end (ca. 53 points), but from the other side, HPLC-DAD is highly integrated and automated and more accessible in many laboratories.

The overall perspective—the whiteness of the methods—indicates that the developed CE-C^4^D approach has a high rank of over 88 points, while—in this comparison—the HPLC-DAD method was marked with 60.8 points. One should notice that the other CE-C^4^D method developed by Saiz et al. [20] is very similar and its lower whiteness value (77.2) is mainly caused by the lack of information on crucial validation parameters.

## 3. Materials and Methods

### 3.1. Chemicals and Reagents

All chemicals used were of analytical grade. Atropine (Atr), scopolamine hydrobromide hydrate (Sco), procaine (IS), acetic acid (≥99.8%), L-histidine (HIS), 2-(*N*-morpholino)ethanesulfonic acid (MES), β-cyclodextrin (β-CD), 2-hydroxypropyl-β-CD (HP-β-CD), 2-hydroxypropyl-γ-CD (HP-γ-CD), 2-hydroxyethyl-β-CD (HE-β-CD), heptakis(2,3,6-tri-O-methyl)-β-CD (TM-β-CD) and magnesium L-lactate were purchased from Sigma-Aldrich (St. Louis, MO, US). Lithium chloride, potassium chloride and calcium chloride hydrate were from Chempur (Toruń, Poland). Methanol was obtained from Honeywell (Charlotte, NC, US). Sodium hydroxide 30% solution was supplied by Avantor Performance Materials S.A. (Gliwice, Poland). Ultrapure water (18.2 MΩ·cm, 3 ppb TOC) was generated in our laboratory in a Milli-Q system by Merck-Millipore (Darmstadt, Germany).

Samples of *Solanaceae* seeds: *Datura metel* (DM), *Hyoscyamus niger* (HN), *Datura stramonium* (DS), *Datura arborea* (DA), *Atropa belladonna* (AB) and *Scopolia lurida* (SL) were purchased (DM, DA, HN, AB) or collected in the Botanic Garden of Jagiellonian University in Kraków, Poland (DS, SL). The plant material was placed in paper bags and stored in a dry and dark place. Few days prior to the extraction, the plant material was ground in an electric grinder.

Samples of pharmaceuticals were the following: eye drop solution (Atropinum sulfuricum, WZF 1%, Polfa Warszawa S.A., Warszawa, Poland) and injection solution (Atropinum sulfuricum, WZF, Polfa Warszawa S.A., Warszawa, Poland).

### 3.2. Preparation of Stock and Standard Solutions

Stock solutions of atropine, scopolamine and procaine (used as IS) at a concentration of 10 mg/mL were prepared in methanol. Working standard solutions at a concentration of 1 mg/mL and 0.5 mg/mL were obtained by diluting the 10 mg/mL stock solutions with methanol. All stock and standard solutions were stored in amber glass vials in the refrigerator for a maximum of 6 months.

For calibration purposes, the solutions at following concentration of Atr and Sco were prepared: 5, 10, 15, 20, 30, 40 and 50 μg/mL. Additionally, each calibrant contained 20 μg/mL of IS and inorganic cations K^+^, Na^+^, Ca^2+^, Mg^2+^, Li^+^ (ca. 2 μg/mL each). The total content of methanol was 35% (*v/v*) to obtain composition similar to the sample matrix.

All BGEs were prepared at least one day before analysis and were used for up to one month after preparation. Every working day, the BGEs were degassed in an ultrasonic bath prior to the analysis. Composition of the used BGEs is described in Section 2.1.1.

### 3.3. Extraction Protocol

The ground plant material (10.0 ± 0.5 mg) was extracted in 1 mL of the extraction solution consisting of methanol and water (ratio 7:3, *v/v*) with the IS at a concentration of 40 µg/mL. The extraction was carried out in an ultrasonic bath for 30 min at 60 °C. After extraction, the extraction vessels were left for 15 min at room temperature. The extracts were then centrifuged for 10 min (10,000 rpm, +5 °C) and the solution was transferred into a 1.5 mL conical vial. Before the measurement, the extracts were diluted twice with ultrapure water. The final sample contained 35% (*v/v*) of methanol.

### 3.4. Instrumentation

A purpose-made CE instrument was built in-house (see Figure 3). The instrument consisted of a positive high-voltage power supply (Spellman UM25P4, Spellman, Pulborough, UK) with two Pt/Ir wire electrodes (of a 0.5 mm outer diameter, 5 cm long). The electrodes were inserted into two 1.5 mL conical vials (Eppendorf AG, Hamburg, Germany) with a hole made in the lid, containing the BGE. A fused-silica capillary (Beckman-Coulter, Brea, CA, USA, i.d. 50 μm, o.d. 365 μm) had a 29.9 cm total length and 18.4 cm effective length (to the detector). A new capillary was rinsed sequentially with methanol (5 min), 1 M NaOH (5 min), 0.1 M NaOH (10 min), ultrapure water (10 min) and BGE (10 min) by vacuum using a 12 ml syringe. The same procedure was applied in the beginning of each working day. The sample injection was performed manually by elevating the injection capillary end to the height of 7 cm for 30 s. After each analysis, the capillary was flushed with BGE for 1.5 min to maintain the reproducibility of migration times. After the last analysis during the working day, the capillary was rinsed with methanol (2 min), ultrapure water (5 min) and air (2 min). The separations were carried out at ambient temperature with the voltage of + 11 kV, applied at the injection side. The detection system was a C^4^D (eDAQ, Deninstone East, Australia). The C^4^D consisted of a C^4^D unit (ER225) and a capillary headstage (ET120). For the optimized BGE, the detector parameters were set as follows: frequency 300 kHz, 60% (12 V) amplitude and headstage gain ON. The equivalent conductivities of Atr and Sco using the developed method are lower than the conductivity of the BGE; thus, the detector signal was inverted to display positive peaks in the electropherograms.

### 3.5. Data Treatment

The PowerChrom (eDAQ, Deninstone East, Australia) software was used for data collection and analysis. This software enabled the display and collection of electropherograms in real time, peak integration and quick data export to MS Excel spreadsheet where further calculations were made. In addition, the C^4^D Profiler V2 (eDAQ, Deninstone East, Australia) software was used to set the optimum operating parameters (frequency, amplitude and headstage gain settings) for the BGE and, thus, to ensure the maximum signal sensitivity for the C^4^D detector. Limits of detection and limits of quantification were determined experimentally by analyzing solutions with decreasing concentration of analytes, as well as a blank sample and visual assessment of the obtained results (see Figure S2 in Supplementary Materials). For LOD, the signal should be distinguishable from the noise (S/N = 3), while for LOQ, the signal-to-noise ratio should be equal or greater than 10. The noise level was calculated as RMS (root mean square) noise.

## 4. Conclusions

In this work, we developed a new CE-C^4^D method for fast analysis of Atr and Sco in plant and pharmaceutical samples. The developed method is characterized by satisfactory precision, recovery and matrix effect on every considered level and the results of the research demonstrate its capability to determine atropine and scopolamine in plant extracts. Moreover, the method may be described as accurate as it was shown in the validation study and testing of pharmaceuticals. The low detection limits established within this study, together with the possibility to separate two similar target compounds strongly supports the statement that the developed method and instrument offers sensitive and selective analyses. The developed method also should be recognized as a promising green analytical chemistry example. Its power, reagent and sample consumption are very low; the toxicity of reagents is minimal and additional hazards may be circumvented by only short training of an operator.

The CE-C^4^D instrument developed within this work is robust and relatively easy in construction. The CE separation module can be reproduced in any lab at low cost (ca. 750 euro, taking into account the most expensive part—the HV power supply) with the cost per analysis lower than 0.3 euro. Together with the commercially available C^4^D detector, the instrument is portable (ca. 6 kg, including a notebook) and it can be run outside the laboratory. However, at the current development stage it requires 110–230 V AC power supply to operate. The drawback of the instrument which has been met during the experiments is the laborious manual operation. Certainly, the next step of the instrument improvement should be integration between Arduino and acquisition software and its hyphenation with a simple autosampler for sequential analysis of multiple samples unsupervised by the operator.

The method was additionally assessed using the RGB model, which enables its comparison with other published approaches. The RGB method algorithm was found to be fast and effective, simultaneously supporting and organizing the discussion on developed method on the canvas of previously published ones. Even if this methodology is affected by the researcher perspective, it is certainly worth applying as it enables one to emphasize positive sides of the method, also outlining the drawbacks and further steps for improvement.

In addition to its simplicity, the CE-C^4^D method shows a potential for discriminative analysis of plant samples based on the observed differences in the concentration of the atropine and scopolamine, as well as matrix components in the tested plant material (seeds of *Solanaceae* plants), and this will be a subject for further investigation.

## Figures and Tables

**Table 1:** Concentration (μg/g) of atropine and scopolamine in *Solanaceae* plants.

Sample ID ^1^	Atropine ^2^	Scopolamine ^2^
DM1	3994 ± 49	2637 ± 26
DM2	4270 ± 20	2677 ± 16
HN1	609.6 ± 21.7	251.2 ± 14.3
HN2	602.4 ± 13.4	214.8 ± 13.7
DS1	985.5 ± 13.1	<LOQ
DS2	1096 ± 13	<LOQ
DA1	467.3 ± 3.0	1827 ± 26
DA2	521.8 ± 4.5	2080 ± 22
AB1	8906 ± 20	ND
AB2	9810 ± 83	ND
SL1	3004 ± 64	1085 ± 17
SL2	3135 ± 60	1122 ± 26

^1^ DM-*D. metel*, HN-*H. niger*, DS-*D. stramonium*, DA-*D. arborea*, AB-*A. belladonna*, SL-*S. lurida*. ^2^ Concentration ± SD, *n* = 3 (μg/g).

**Table 2:** Validation parameters for atropine and scopolamine.

Parameter	Atropine	Scopolamine
Linearity (μg/mL)	5–50	5–50
Slope	0.027	0.032
Intercept	−0.034	−0.023
R-squared	0.999	0.996
LOD (μg/mL)	0.5	0.5
LOQ (μg/mL)	1.5	1.5
Precision ^1^, CV (%):		
5 μg/mL	1.88	1.66
20 μg/mL	2.08	1.07
50 μg/mL	1.18	1.73
Intermediate precision ^1^, CV (%):		
5 μg/mL	1.69	2.28
20 μg/mL	2.13	2.77
50 μg/mL	1.16	1.59
Recovery, RE (%):		
+12.5 μg/mL	102.7	101.0
+25 μg/mL	101.3	101.8
Matrix effect, ME (%):		
+12.5 μg/mL	93.56	92.99
+25 μg/mL	94.10	94.23

^1^ *n* = 5 for precision; *n* = 10 for intermediate precision.

## Data Availability

Not applicable.

## Supplementary Materials

The following are available online, Figure S1: Effect of shortening effective length of the capillary and increasing the applied voltage on the separation, Figure S2: Experimental confirmation of the determined LOD and LOQ values, Table S1: Chemical characteristics of prepared BGEs. The excel spreadsheet with the assessment of analytical methods using the RGB scale.

## Sample Availability

Samples of the compounds are not available from the authors.
