# Supplementary material for: Development of CE-C4D Method for Determination Tropane Alkaloids
Source: Molecules. 2021 Sep 22;26(19):5749. doi: 10.3390/molecules26195749 (PMC8510007; doi:10.3390/molecules26195749)
Supplement: Supplementary file 1 [file molecules-26-05749-s001.zip › Supplementary-rev.pdf]

Supplementary

Table S1. Chemical characteristics of prepared BGEs.

Abbreviation	pH	Composition	Conductivity[1] (S/m)	Ionic strength[1] (mM)	Buffer capacity[1] (mM)
A-2.5	2.5	0.5 M acetic acid	0.120	3.13	14.4
A-3.1	3.1	0.025 M acetic acid	0.026	0.673	3.06
HA-4.1	4.1	0.005 M HIS, 0.025 M acetic acid	0.034	5.12	9.73
HM-6.1	6.1	0.02 M HIS, 0.02 M MES	0.047	10.2	23.1

[1] Calculated with PeakMaster 6.0f8

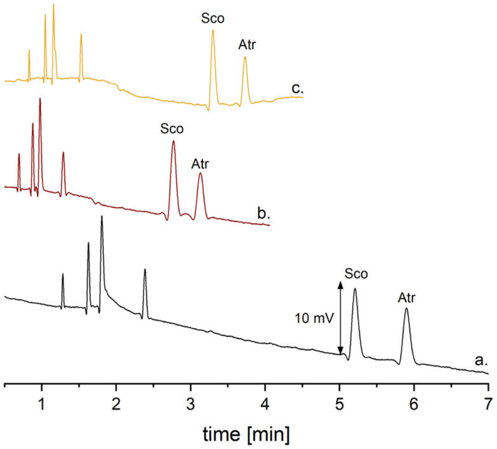

Figure S1. Effect of **(b)** shortening the effective length of the capillary (from 18.4 cm to 9.5 cm; R ~ 2.7); **(c)** increasing the applied voltage (from +11 kV to +15 kV; R ~ 4.1). **(a)** Electropherogram obtained in the initial conditions: effective capillary length – 18.4 cm, applied voltage: + 11 kV (R ~ 4.1). Sample: 25 µg/mL Atr and Sco and ~ 2 µg/mL inorganic cations (K^+, Na^+, Ca^{2+}, Mg^{2+}, Li^+). Injection was performed hydrodynamically (siphoning) by elevating the injection capillary end to the height of 7 cm for 30 s.

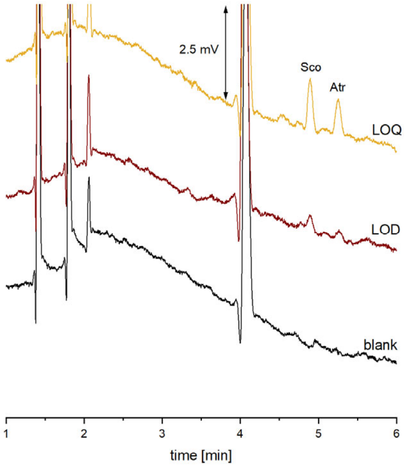

Figure S2. Experimental confirmation of the determined LOD and LOQ values. LOD and LOQ for atropine and scopolamine were found to be the same and they were 0.5 and 1.5 µg/mL, respectively.
